# Examining feed preference of different pellet formulations for application to automated milking systems

**DOI:** 10.3168/jdsc.2022-0318

**Published:** 2023-02-09

**Authors:** A.L. Carroll, K.K. Buse, J.D. Stypinski, C.J.R. Jenkins, P.J. Kononoff

**Affiliations:** 1 Department of Animal Science, University of Nebraska, Lincoln 68503; 2 Standard Dairy Consultants, Omaha, NE 68144

## Abstract

Feed is often offered to a cow in the milking unit of an automated milking system. This offering provides nutrients but also acts as a reward to the cow for entering the unit. To complement the partial total mixed ration and to enable handling, flow, and delivery within this mechanized system, this offering is usually a mix of feeds that are combined and manufactured into a feed pellet. The objective of this experiment was to compare 4 different pelleting formulation strategies and measure the effects of feed preference in lactating Jersey cattle. To test the objective, a taste preference experiment was conducted with 8 multiparous lactating Jersey cattle (289 ± 25.3 d in milk, 26.0 ± 2.45 kg of milk yield, 19.36 ± 1.29 kg of dry matter intake). Four formulation strategies were tested including (1) a pellet containing feeds commonly included in the concentrate mixture of a total mixed ration, including 43.1% corn grain, 26.3% dried distillers grains, 3.18% soybean meal, and 5.6% vitamin and mineral premix (CMIX), (2) a pellet of dry corn gluten feed (CGF), (3) a pellet including feedstuffs that are considered to be highly palatable (53.2% wheat middling, 15.7% dried corn distillers grains and solubles, 15.2% cane molasses, and 1.81% oregano (FLVR), and (4) a high-energy pellet (ENG) consisting of 61% corn grain and 26.2% wheat middlings. Cows were offered 0.50 kg of each in a randomized arrangement within the feed bunk for 1 h or until the feed was fully consumed. According to the procedure, cows were offered all 4 treatments for the first 4 d, then the most preferred feed for each cow was removed, and the remaining 3 feeds were offered for 3 d. The process was repeated for the last 2 d. Feed preference was ranked from 1 to 4 with 1 being the most preferred and 4 the least. The resulting preference ranking was CGF (1.25 ± 0.463), FLVR (2.5 ± 0.926), CMIX (2.88 ± 0.835), and ENG (3.13 ± 0.991). These results were subsequently examined utilizing the Plackett-Luce analysis to examine the probability animals would choose a given pellet first based on the current data set. The analysis determined probabilities of first choice as 78.6 ± 0.601% CGF, 9.38 ± 0.438% FLVR, 4.94 ± 0.453% ENG, and 7.11 ± 0.439% CMIX. A *Z*-test was also conducted to determine if the percentage a treatment will be chosen first differed from the mean value of no preference at 25%. Corn gluten feed and ENG differed from the mean value while no difference was observed for FLVR and CMIX. Results suggest that animals exhibit a high degree of preference for CGF pellets and that this preference is greater than pellets containing other feed ingredients. Alternatively, cows appeared to exhibit the lowest preference for a high-energy pellet containing mostly corn and wheat middlings.

## Highlights

• Examining feed preference of 4 pellet formulation strategies.
• Pellets: high flavor, corn gluten feed, concentrate mix, and high energy.
• Animals preferred corn gluten feed pellets.

## Graphical Abstract

**Figure:**
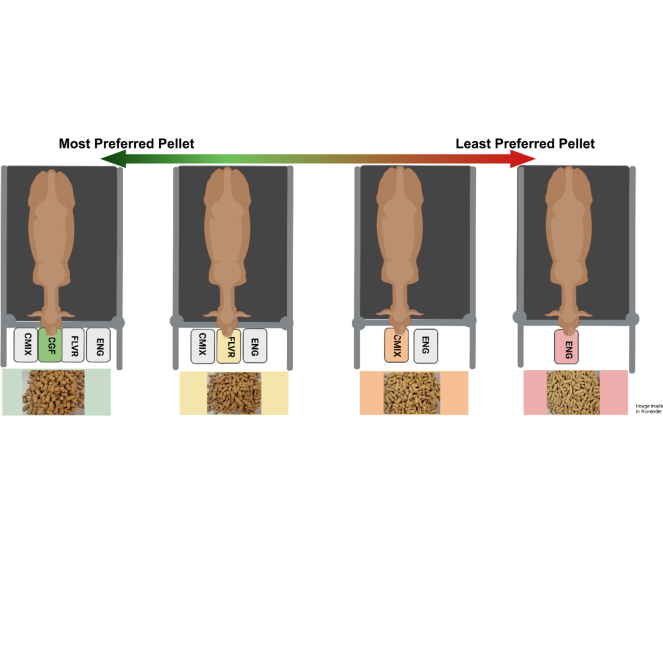
**Summary:** In recent years, the dairy industry has been shifting toward the utilization of automated milking systems. As such, pelleted feeds are often used to incentivize cows to enter the milking box; however, data comparing different pelleting strategies are currently limited. The current experiment examined different pelleting formulation on feed preference of lactating Jersey cattle. Four pelleting formulations were compared in the taste preference experiment, including a pellet containing ingredients commonly found in dairy concentrate mixes (CMIX), a dry corn gluten feed (CGF) pellet, a pellet containing ingredients previously described to be palatable with oregano leaf (FLVR), and a high-energy pellet (ENG). Cow preference ranking from greatest to least preferred were CGF, FLVR, CMIX, and ENG pellet. Based upon Placket-Luce analysis, we conclude cows choose CGF pellets 78% of the time. These results suggest that animals prefer CGF pellets relative to those pellets containing what are often considered highly palatable ingredients, and exhibited lowered preference for pellets containing higher concentrations of starch.

There are approximately 50,000 automated milking systems (**AMS**) worldwide, and AMS is a sector within dairy production that continues to grow in popularity (Maculan and Lopes, 2016). Motivating animals to enter the milking unit by offering palatable and usually pelleted mixes of ingredients meets the need of animals to consume nutrients and increases the number of animal visits to the robot, milking frequency, and production (Migliorati et al., 2009). Although reduced visits to the robot could be a function of pellet preference and pellet quantity, experiments have suggested that the quantity of pellets does not affect the number of visits to the robot or required fetching (Halachmi et al., 2005; Bach et al., 2007). This could be because animals are more motivated by feed preference alone and not the amount of pellet offered. Therefore, inclusion of preferred ingredients may have a greater impact. For example, increasing the inclusion of molasses or other highly palatable ingredients has been linked to increases in the number of voluntary visits and decreases in fetching (Rodenburg and Wheeler, 2002). To differentiate the effects of pellet preference and quantity, an experiment by Migliorati et al. (2005) tested a fenugreek (*Trigonella foenum-graecum*) flavoring and pellet allowance. Results of this experiment indicated that the amount of pellet within the AMS had no effect on visits per day; however, fenugreek did increase the number of visits and decreased the interval between visits while numerically increasing the milk yield. In general, palatability is believed to be a factor that influences visits with milking to the AMS (Madsen et al., 2010) and could have practical implications on feed cost related to pellet formulation strategies, but data are lacking in comparing differing pellet formulation strategies on feed preference. The objective of this experiment was to examine preference of 4 pelleting formulation strategies. We hypothesized that the most preferred feed would be the pellet designed with feedstuffs indicated to be palatable in dairy cattle.

Before conducting the experiment, procedures using animals were approved by the University of Nebraska–Lincoln Institutional Animal Care and Use Committee. Eight multiparous lactating Jersey cattle (289 ± 25.3 DIM, 26.0 ± 2.45 kg of milk yield, 19.36 ± 1.29 kg of DMI) were utilized for the taste preference experiment in a manner described by Erickson et al. (2004). This study was not conducted in an AMS; rather, cows were housed in tie-stalls with continuous access to water and fed 85% of the previous week's average intake once daily at 1000 h. After milking at 1800 h and returning from the exercise pen at 1930 h, feed was removed from the individual feed bunks and animals were offered 0.50 kg of each pellet treatment in a randomized arrangement and separated within 12 inch × 16 inch (30.48 cm × 40.64 cm) plastic tubs. These were offered for 60 min or until a single treatment was fully consumed. Feeds were offered for 9 d total as described with all 4 pellets allocated from d 1 to 4. After the initial feeding period, the most preferred feed was removed, and 3 feeds were offered for 3 d. Then, the process was repeated for 2 d. Treatments were ranked sequentially with 1 being most preferred and 4 being least. This is the same way in which they were removed during the experiment. The 4 pellets that were examined included a single pellet of dry corn gluten feed (**CGF**); a pellet including feedstuffs that are considered to be highly palatable [53.2% wheat middling, 15.7% dried distillers grains, 15.2% cane molasses, and 1.81% oregano (**FLVR**)]; a high-energy pellet (**ENG**) consisting of 61% corn grain and 26.2% wheat middlings; and a pellet containing feeds commonly included in the concentrate mixture of a TMR including 43.1% corn grain, 26.3% dried distillers grains, 3.18% soybean meal, and 5.6% vitamin and mineral premix (**CMIX**). The CGF pellet originated from ADM (Cedar Rapids, IA), whereas the other treatments were custom pelleted through Cooper Specialty Feeds (Union, NE). The CMIX was included as a treatment because we observed that a similar formulation and offering was provided on a commercial AMS dairy in Nebraska.

Chemical composition of each pellet was determined. To do so, pellets were first ground through a 1-mm screen (Wiley Mill; Arthur A. Thomas Co.) and then analyzed for DM (AOAC International, 2000), N (Leco FP-528 N Combustion Analyzer; Leco Corp.), amylase-treated NDF (**aNDF**; Van Soest et al., 1991), NDF with sodium sulfite and α-amylase corrected for ash contamination (**aNDFom**; Van Soest et al., 1991), starch (Hall, 2009), and total fatty acids (Sukhija and Palmquist, 1988) by Cumberland Valley Analytical Services Inc. (Hagerstown, MD). Samples were also analyzed for ash according to method 942.05 (AOAC International, 2000) at the Ruminant Nutrition Laboratory at the University of Nebraska–Lincoln. Pellet hardness was determined according to Berends et al. (2018), where pellet hardness was measured as the compression force (kg) required to fragment a pellet into small particles.

Treatment rankings were analyzed for mean and standard deviation using the PROC CORR function of SAS (version 9.4; SAS Institute Inc.). Data were also analyzed with the Plackett-Luce Model in R (version 1.3.1093; https://www.r-project.org/) to estimate the probability an examined pellet would be chosen first based on the rankings from the current data set. A Z-test was conducted in R on the probabilities to determine if the probability differed from the probability of no preference at 25% (100/4; Fligner and Verducci, 1988). Significance was declared with a *P*-value ≤0.05.

The aim of this experiment was to examine the preference of 4 different pelleting formulation strategies on feed preference. Table 1 lists the pellet formulation strategies and chemical composition of the 4 experimental pellets. As expected, 4 pellet treatments differed in chemical composition, but the objective of the current experiment was not to test the impacts of chemical composition or nutrient concentration on preference. The authors also recognize that in AMS, nutrition pellets are generally formulated to balance the nutritional needs of the animal with the partial total mixed ration, but in the current experiment, the partial total mixed ration was the same for animals consuming all treatments. Although nutrient provision and incentive to enter the AMS systems are goals of practical AMS diet formulation, we recognize that since a small quantity was offered relative to on-farm AMS systems and TMR access was limited to 85% of the previous week's intake, we believe we did not affect nutrient intake to an extent that physiological factors would affect preference behavior. As expected, CGF contained more CP (21.0%) than CMIX, FLVR, and ENG (18.6, 17.7, and 14.2%, respectively). Amylase-treated NDF and aNDFom were similar for CGF and FLVR (31.7 and 31.2 vs. 31.5 and 31.1), likely as a function of the approximate 38.7% aNDF content of wheat middlings in the FLVR treatment (NASEM, 2021). These concentrations were increased relative to the CMIX pellet at 24.1% and the ENG at 19.8%, and were likely due to the dilution of fiber-containing components with mineral inclusion in the CMIX as well as increased corn grain content in both CMIX and ENG. Increasing the corn grain content within CMIX and ENG had a subsequent impact on the starch content increasing the concentrations to 49.3 and 31.5% starch relative to those of CGF and FLVR at 15.6 and 21.3%. Also, ash content was increased with the CGF pellet and the CMIX pellet at 7.84% and 5.38%. These values fall in line with the NASEM (2021) ash values for corn gluten feed, and we formulated the CMIX pellet to contain vitamin and minerals.

**Table 1:** Ingredient inclusion and chemical composition of experimental pellets (% of pellet DM, unless otherwise indicated)^1^, ^2^

Item	CMIX	CGF	FLVR	ENG
Ingredient				
Corn, rolled	43.1	—	15.7	61.0
Dried corn distillers grains and solubles	26.3	—	14.2	8.89
Wheat middlings	13.8	—	53.2	26.2
Dry corn gluten feed pellet	—	100	—	—
Soybean meal	3.18	—	—	—
Dry molasses	7.10	—	—	—
Liquid cane molasses	—	—	15.2	2.90
Dextrose	—	—	—	—
Oregano	—	—	1.81	—
Corn oil	0.93	—	—	1.00
Vitamin premix^3^	0.09	—	—	—
Trace mineral premix^4^	0.06	—	—	—
Calcium carbonate	1.30	—	—	—
Magnesium oxide	0.49	—	—	—
Sodium sesquicarbonate	1.88	—	—	—
Salt, white	0.86	—	—	—
Whey permeate	0.92	—	—	—
Chemical composition (% of DM)				
DM (%, as is)	85.2	89.7	82.4	86.3
CP	18.6	21.0	17.7	14.2
aNDF^5^	24.1	31.7	31.5	19.8
aNDFom^6^	23.8	31.2	31.1	19.3
Starch	31.5	15.6	21.3	49.3
Total fatty acids	5.59	3.66	5.21	5.65
Ash	5.38	7.84	4.97	2.22
Hardness, kg of force (SD)^7^	7.9 (2.94)	20.8 (7.05)	12.9 (3.60)	10.8 (2.84)

1 Pellet formulations: CGF = corn gluten pellets; FLVR = high-palatability oregano; ENG = energy balance; and CMIX = typical pellet containing vitamins and minerals.

2 Diet composition (% diet DM): corn silage 28.7%, alfalfa hay 28.7%, fine ground corn 25.0%, expellers soybean meal 7.78%, soybean meal 0.83%, blood meal 0.67%, rumen-protected methionine 0.08%, beet molasses 1.81%, fat supplement 3.11%, ground soybean hulls 1.30%, salt 0.47%, sodium bicarbonate 0.34%, calcium carbonate 0.21%, magnesium oxide 0.59%, dicalcium phosphate 0.28%, vitamin premix 0.04%, and trace mineral premix 0.04%.

3 Formulated to supply approximately 1,133.79 kIU/d vitamin A, 181.41 kIU/d vitamin D, and 53.51 IU/d vitamin E in total rations.

4 Formulated to supply approximately 2,000 mg/kg Co, 20,000 mg/kg Cu, 2,000 mg/kg I, 5 mg/kg Fe, 100,000 mg/kg Mn, 625 mg/kg Se, and 15 mg/kg Zn in total rations.

5 Van Soest et al. (1991) using α-amylase and sodium sulfite. aNDF = amylase-treated NDF.

6 Amylase-treated NDF on an organic matter basis.

7 Pellet hardness was determined as the compression force (kg) required to fragment a pellet into small particles.

Feed preference was ranked, with 1 being most preferred and 4 being the least, and then analyzed for mean and standard deviation. The resulting preference ranking for CGF, FLVR, CMIX, and ENG was 1.25 ± 0.463, 2.50 ± 0.926, 2.88 ± 0.835, and 3.13 ± 0.991, respectively (Table 2). Based on the preference ranking, a pellet containing a single feed ingredient being observed to be most palatable in the present study was surprising. Dry corn gluten feed contains a portion of steep water, distillers solubles, and corn bran, all which undergo a heating process during drying as well as steam extrusion during pelleting. During heating, Maillard reactions occur where available carbohydrate complexes react with AA, creating aromatic compounds, such as furosine, that may contribute to the sweet sensory perception (Jo et al., 2018). While the sweet aroma may have been enticing to cows, the sweet taste was likely not the primary driver; the FLVR contained molasses, and as a result, more sugar (NRC, 2001). Similar to sweet taste preference in dairy cattle, umami tastes are highly preferred, as displayed by preference for monosodium glutamate (Nombekela et al., 1994; Roura and Navarro, 2018). Glutamate is a primary, taste-active umami flavor in food additives, such as monosodium glutamate, formed through fermentation of starch sources similar to dry and wet milling practices, that produce corn gluten feed (Nombekela et al., 1994). Therefore, we speculate that the umami flavor is likely present in CGF due to the addition and condensation of distillers solubles from starch fermentation and may have been preferable to cows. It should be noted that it is possible that a factor affecting preference of CGF was pellet size (Beauchemin, 1991). In the case of the current study, pellet size was different between treatments. Specifically, the CGF was manufactured and pelleted at the wet corn milling plant, resulting in a pellet width of 9.53 mm, whereas the pellet width of remaining treatments was 6.35 mm. The CGF treatment was designed to be a simple, low-cost alternative to the remaining treatments that were strategically formulated. It is possible that pellet length played a role because it has been observed to be associated with feed intake (Beauchemin, 1991), and a linear increase in eating rate with increasing pellet length has also been observed Spörndly and Åsberg (2006). When interpreting data related to feed preference, one animal factor to consider is previous exposure to feeds. All animals on this experiment originated from a commercial farm and had previously been fed both wet and dry corn gluten feed within their lifetime. Although animals had not been exposed to oregano leaf, they had been previously fed DDGS, corn, and molasses, which made up 45.1% of the pellet. Increasing inclusion of corn gluten feed has been observed to increase DMI in lactating cattle when replacing other concentrates including corn grain, soybean meal, and soybean hulls (Mullins et al., 2010). This supports the notion that although previous exposure could influence preference, other factors likely contribute to the drive to consume the CGF pellet.

**Table 2:** Preference scores of 4 different pelleting strategies in lactating Jersey cattle

Item	Treatment^1^			
	CGF	FLVR	ENG	CMIX
Cow ID				
1	1	2	4	3
2	2	3	1	3
3	1	3	3	2
4	1	2	3	4
5	1	3	4	2
6	2	1	3	4
7	1	4	3	2
8	1	2	4	3
Sum	10	20	25	23
Mean	1.25	2.5	3.13	2.8
SD	0.463	0.926	0.991	0.835

1 Rank of pellet formulations fed at 0.5 kg per treatment with 1 as most preferred and 4 as least preferred. CGF = corn gluten pellets; FLVR = high-palatability oregano; ENG = energy balance containing 20% of the energy required for an animal milking 32.7 kg/d and consuming 24.2 kg/d DMI; and CMIX = typical pellet containing vitamins and minerals.

Table 3 displays the probability an animal will choose a pellet based on the observations made during the preference test and if that probability differs from a mean value of no choice at 25% (Erickson et al., 2004). The probability of first choice for the CGF pellet was observed to be 78.6 ± 0.601%, which was different from the mean value of no choice at 25%. The second and third choices averaged 9.38 ± 0.438% FLVR and 7.11 ± 0.439% CMIX and did not differ (*P* > 0.19) from the mean values of 25%. The FLVR pellet was likely preferred over the CMIX as the addition of oregano to feed has been previously indicated to improve preference of hydrolyzed feather meal; feather meal has historically been considered an unpalatable feed ingredient (Buse et al., 2021). Another contributing factor in the observed preference ranking is that animals prefer pellets over feeds in the form of a meal (Krogstad et al., 2021). Therefore, the increased fines caused by vitamin and mineral inclusion in the CMIX pellet containing 5.38% ash decreased the pellet hardness. This is further supported by the observation that pellet hardness decreased approximately 62% from 20.8 ± 7.05 kg in the CGF to 7.9 ± 2.94 kg in the CMIX and it is possible this may have negatively affected feed preference. Based on the taste preference experiment, we conclude that animals would choose the ENG pellet 4.94 ± 0.453% of the time and this proportion was significantly (*P* = 0.04) lower than the mean value of no choice at 25%. The ENG pellet within the current experiment contained similar base ingredients to that of the CMIX and FLVR but an increased proportion of corn grain containing starch. Unlike humans, cattle contain no salivary amylase, which converts dietary starch into sugar (McDougall, 1948). As such, the starch contained within the pellet would not be enzymatically degraded to sugars, which then could be perceived by type II sensory cells on the tongue (Lee and Owyang, 2017). Also, animals consumed 85% of the previous week's intake; therefore, the increased energy content of the ENG pellet was not a driver in feed preference in the current experiment. Thus, we speculate that CMIX contained a less desirable flavor relative to all other treatments.

**Table 3:** Probability animals will choose a given pelleting strategy first, based upon the preference of lactating Jersey cattle

Treatment^1^	μ^2^	SE^3^	Z^4^	*P*^5^
CGF	78.6	0.601	3.04	<0.01
FLVR	9.38	0.438	−0.69	0.49
ENG	4.94	0.453	−2.08	0.04
CMIX	7.11	0.439	−1.32	0.19

1 Pellet formulations: CGF = corn gluten pellets; FLVR = high-palatability oregano; ENG = energy balance; and CMIX = typical pellet containing vitamins and minerals.

2 μ = the estimated percentage chance a diet will be chosen when all treatments are presented.

3 SE = standard error for the percentage chance a treatment will be chosen first.

4 Z = the percentage chance that a treatment being chosen first is different from the percentage of no choice at 25%.

5 The *P*-value indicates that the preference value differs from the percentage chance an animal would choose 1 of the 4 feeds at random (25%).

The aim of this experiment was to examine 4 different pelleting strategies for preference in lactating Jersey cattle. Results suggest that animals exhibit a high degree of preference for CGF pellets. Alternatively, cows appeared to exhibit lowest preference for a pellet containing predominantly corn and wheat middlings. Overall, little work has aimed to describe feed preference in dairy cattle although it has direct implications within AMS systems, as previously described. Further exploration on this topic could expand our ability to utilize target chemical compounds within palatable ingredients to improve intake of nutritious yet more unpalatable feeds (Buse et al., 2021). Also, considering feed preference in pellet formulation strategies may aid producers in designing pellets that consider both cost and feed preference. Therefore, further research is needed in preference to define compounds found in feeds as well as describe their influence on preference in cows.
